# Supplementary material for: Infiltrating lipid-rich macrophage subpopulations identified as a regulator of increasing prostate size in human benign prostatic hyperplasia
Source: Front Immunol. 2025 Jan 10;15:1494476. doi: 10.3389/fimmu.2024.1494476 (PMC11757139; doi:10.3389/fimmu.2024.1494476)
Supplement: Supplementary file 2 [file Presentation1.pdf]

Supplementary Information

"Infiltrating lipid-rich macrophage subpopulations identified as a regulator of increasing prostate size in human benign prostatic hyperplasia"

This supplementary information document contains 12 supplementary figures and 3 supplementary tables.

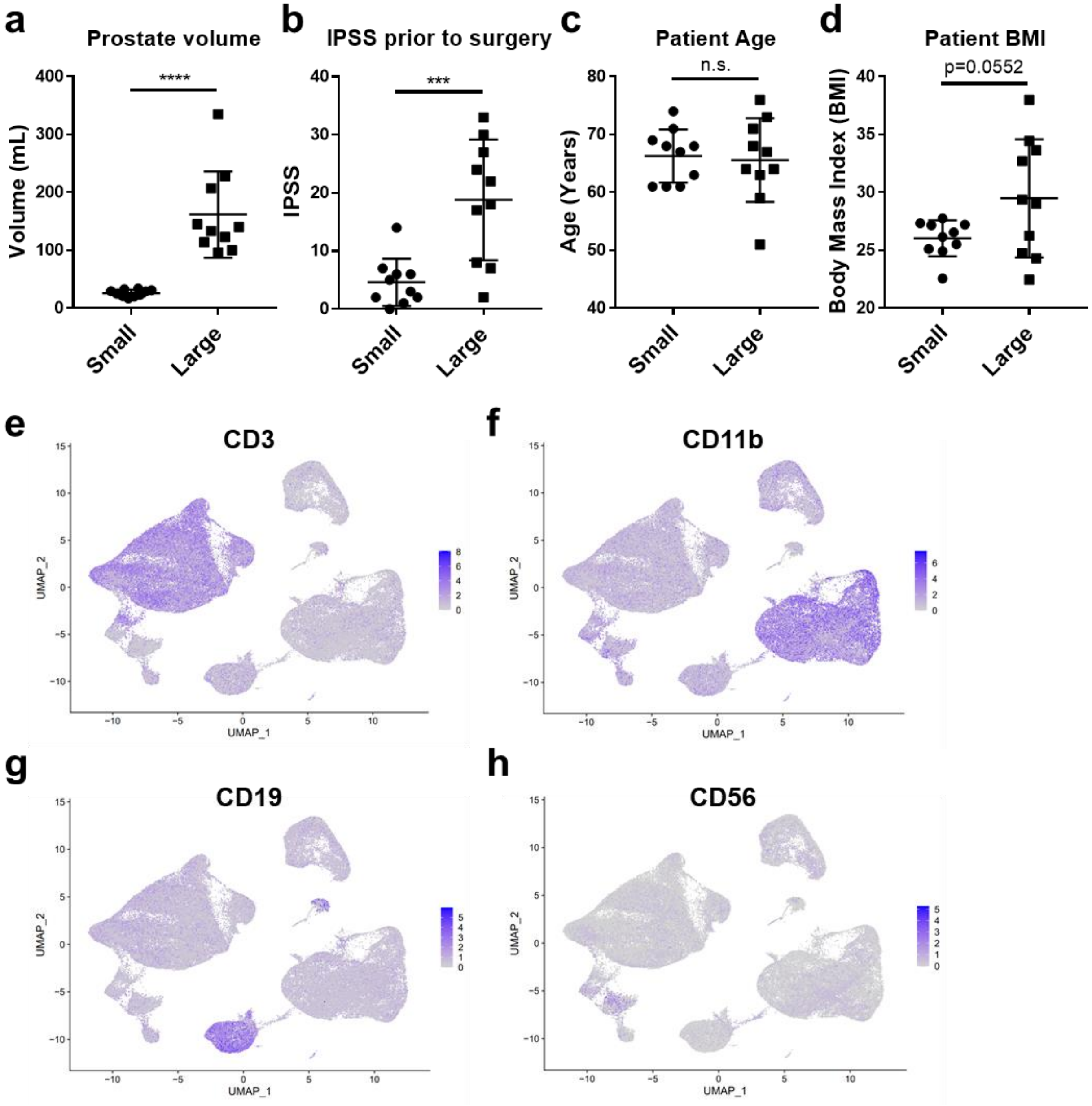

Supplementary Figure S1. scRNA-seq general patient characteristics and identification of major CD45+ cell populations. a) Prostate volume estimation by TRUS, MRI, or CT scan (****p<0.0001, d=2.57), **b)** patient IPSS prior to surgery (***p=0.0008, d=1.80), **c)** patient age

at surgery (p=0.7987, d=0.12), and **d)** patient BMI prior to surgery (p=0.0552, d=0.92) is shown for n=10 patients with small prostates and n=10 patients with large prostates. Statistical analysis was performed using a Mann Whitney U test in (a) and a two-tailed unpaired t-test in (b-d). All patients are biologically independent. Error bars represent the mean +/- SD for each graph. n.s.=not significant. **e-h)** CITE-seq analysis was conducted on a subset of the 20 patient samples and allowed for the visualization of protein expression in single cells. Feature plots highlight protein expression of e) CD3 (T cells), f) CD11b (myeloid cells), g) CD19 (B cells), and h) CD56 (NK cells), where blue color indicates positive expression and gray color indicates no detectable expression of the indicated protein.

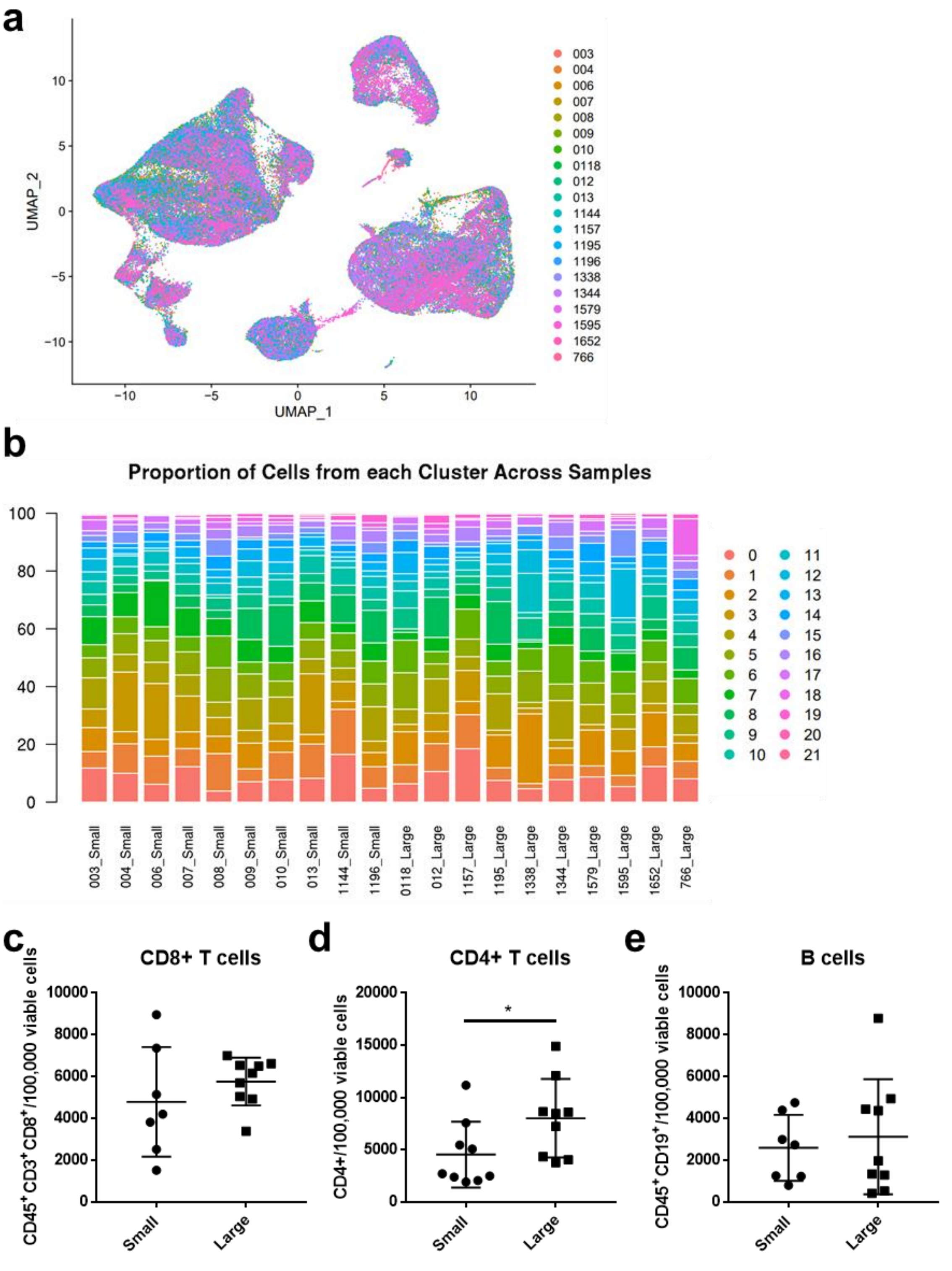

Supplementary Figure S2. Leukocyte infiltration in human prostate TZ tissue on a per-patient basis. Analysis of CD45+ leukocyte infiltration was conducted on n=10 small and n=10 large prostate tissues using flow cytometry and scRNA-seq analysis. **a)** UMAP plot of 100,459 BPH-associated immune cells, colored to highlight cells from individual patients. **b)** Graph

indicates the proportion of cells in each CD45+ scRNA-seq cluster in individual patients (n=20). Colors indicate each cluster identity. **c-e)** Flow cytometry analysis of digested prostate TZ from a subset of the scRNA-seq patient cohort (n=7-9 per group). Graphs show the mean +/- SD of c) CD45+CD3+CD8+ T cells/100,000 viable cells (p=0.3319, d=0.51) using a two-tailed unpaired t-test, d) CD45+CD3+CD4+ T cells/100,000 viable cells (p=0.040, d=1.00) using a Mann-Whitney U test, and e) CD45+CD19+ B cells/100,000 viable cells (p=0.6593, d=0.23) using a two-tailed unpaired t-test. Zombie violet negative events were gated as viable cells.

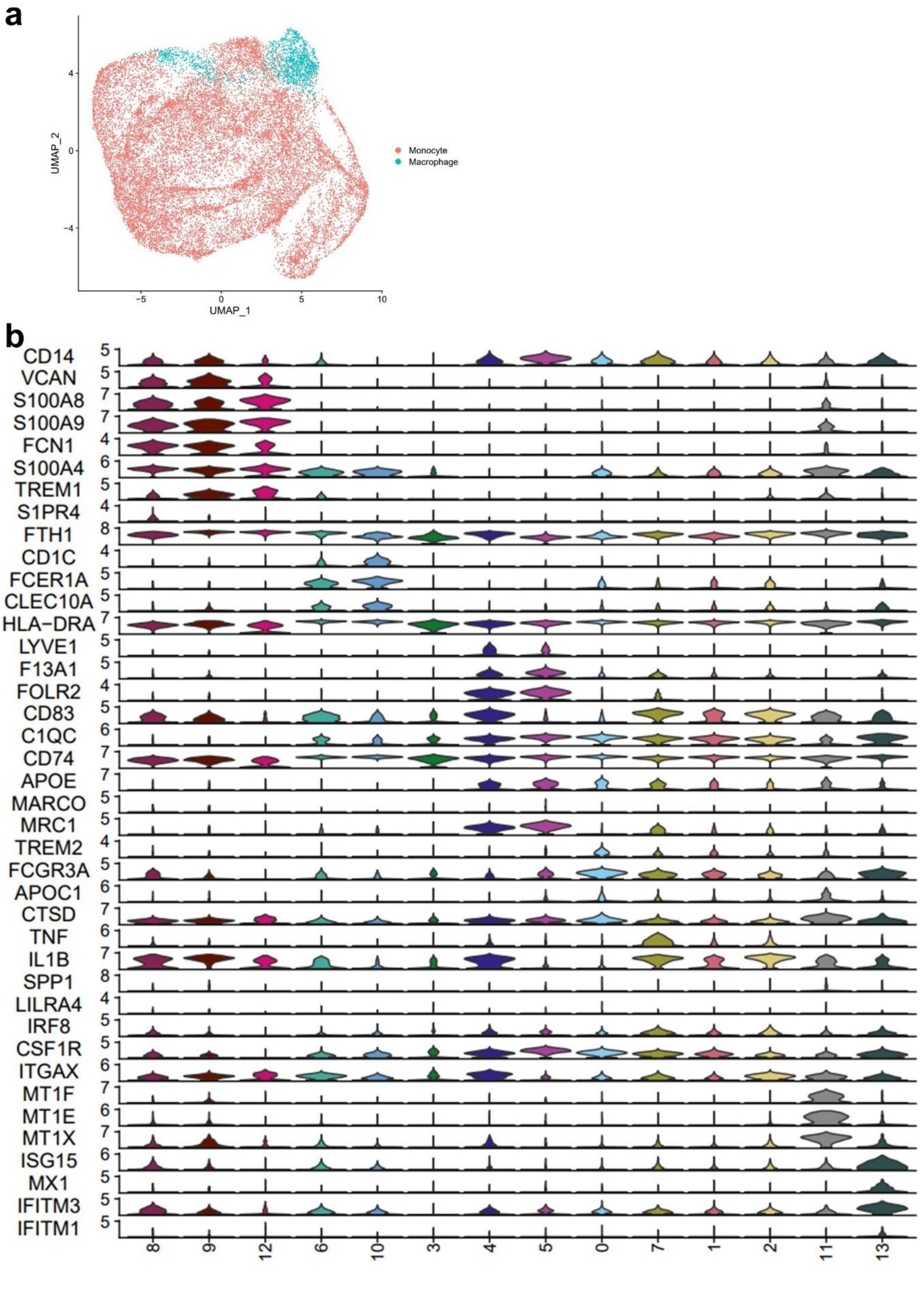
a
UMAP_2
UMAP_1
4
0
−4
−5
0
5
10
Monocyte
Macrophage
b
CD14 5
VCAN 5
S100A8 7
S100A9 7
FCN1 4
S100A4 6
TREM1 5
S1PR4 4
FTH1 8
CD1C 4
FCER1A 5
CLEC10A 5
HLA−DRA 7
LYVE1 5
F13A1 5
FOLR2 4
CD83 5
C1QC 6
CD74 7
APOE 7
MARCO 5
MRC1 5
TREM2 4
FCGR3A 5
APOC1 6
CTSD 7
TNF 6
IL1B 7
SPP1 8
LILRA4 4
IRF8 5
CSF1R 5
ITGAX 6
MT1F 7
MT1E 6
MT1X 7
ISG15 6
MX1 5
IFITM3 5
IFITM1 5
8
9
12
6
10
3
4
5
0
7
1
2
11
13

Supplementary Figure S3. Identification of myeloid cell subpopulations. Myeloid cells were evaluated via subclustering analysis. a) UMAP plot indicating the myeloid cell designations using singleR, where individual cells are labeled as monocytes (pink) or macrophages (blue). **b)** Stacked violin plots of marker genes used to identify myeloid cell subclusters in Figure 2. Clusters are arranged based on proposed similarity.

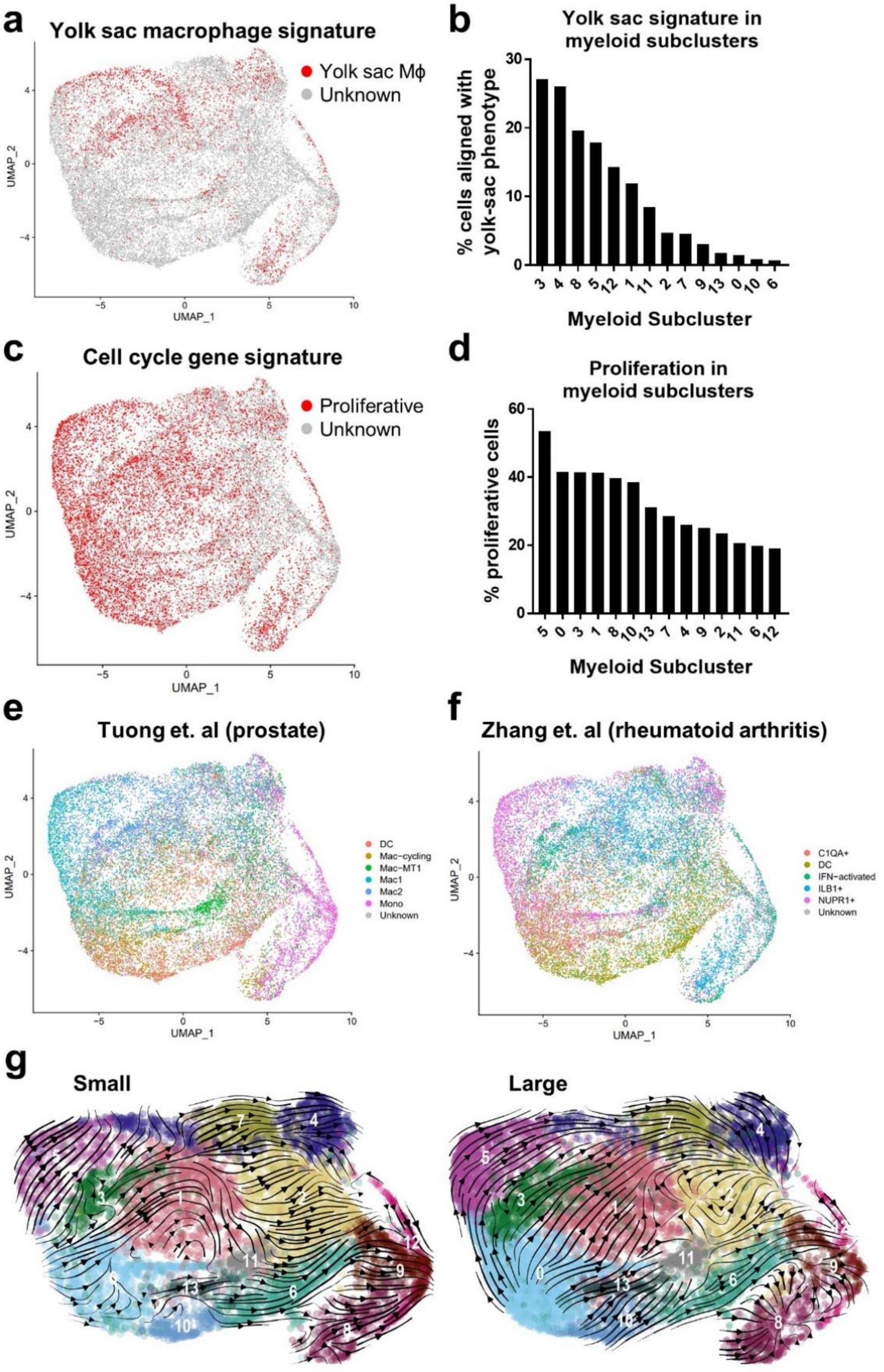

Supplementary Figure S4. Additional information relating to myeloid cell subclustering. a-b) Evaluation of myeloid cell subclustering to determine cells that are transcriptionally similar to yolk sac-derived macrophages was determined via training a classifier using Garnett. **a)** Feature

plot indicates cells that express the transcriptional profile of yolk sac macrophages in red. Other cells with unknown identity are labeled gray. **b)** Graph indicates the percentage of cells in each myeloid subcluster that were identified in (a) as being similar to yolk-sac derived macrophages. **c-d)** Evaluation of proliferating BPH myeloid cells was determined via training a classifier using Garnett based on expression of cell cycle genes. **c)** Feature plot indicates expression of cell cycle genes, where a higher red intensity indicates greater expression of cell cycle (proliferation) genes. **d)** Graph indicates the percentage of proliferative cells in each myeloid subcluster based on alignment of cell cycle genes in (c). **e-f)** BPH myeloid cells were assigned to defined to subpopulations in e) prostate tissue and f) synovial tissue from rheumatoid arthritis via Garnett-trained classifiers. The plots indicate the assigned identities projected onto the myeloid subclustering UMAP plot. **g)** RNA velocity analysis was conducted to evaluate cellular trajectories within the myeloid subclustering analysis. The dynamical model of the velocity stream is projected onto the UMAP plot for each small (n=10) and large (n=10) samples.

Cluster 0 (*TREM2*+ Mϕs) PPAR signaling pathway

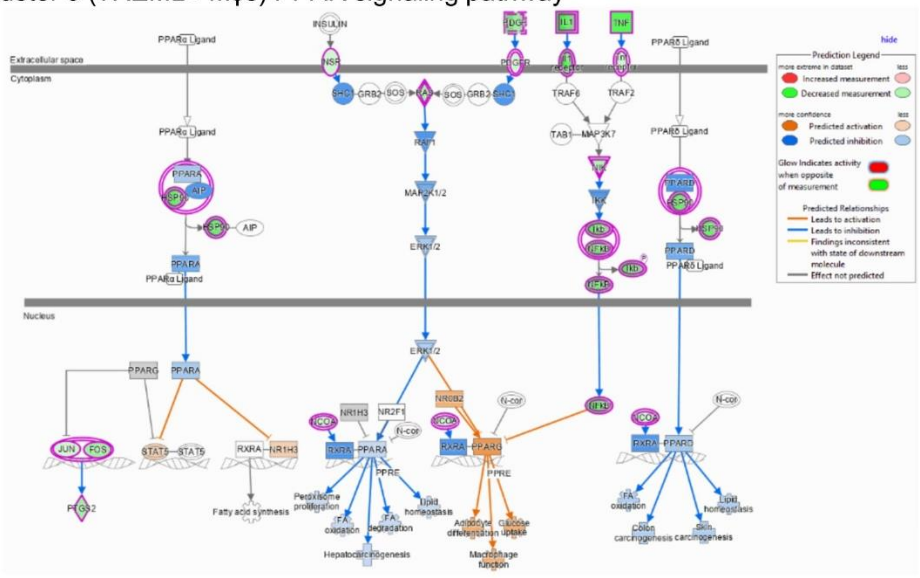

Supplementary Figure S5. TREM2+ macrophages have activation of the PPAR signaling pathway. IPA analysis was used to evaluate signaling pathways based on differential gene expression of each subcluster versus all other subclusters. The PPAR signaling pathway for TREM2+macrophage subcluster 0 and predicted activation (orange) or inhibition (blue) is shown.

Cluster 5 (*MARCO*+ Mϕs) PPAR signaling pathway

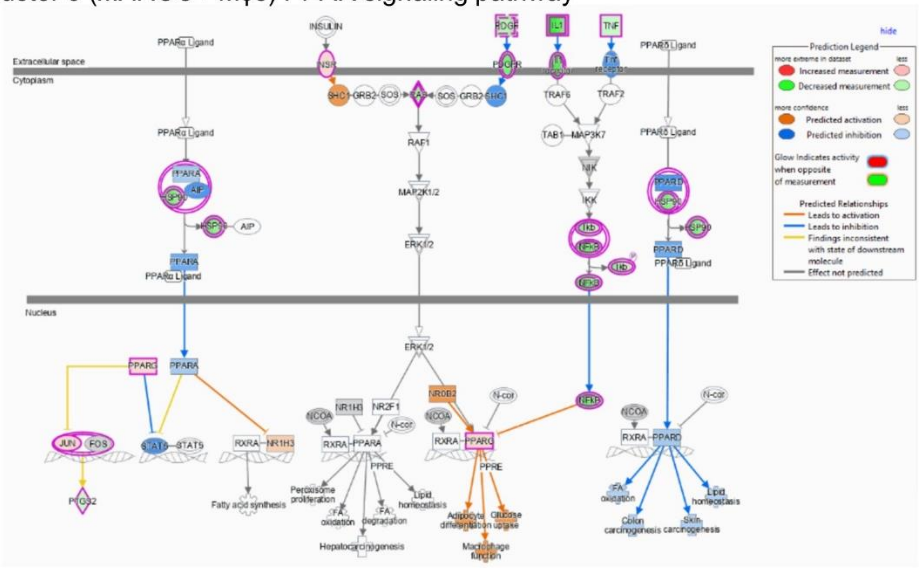

Supplementary Figure S6. MARCO+ macrophages have activation of the PPAR signaling pathway. IPA analysis was used to evaluate signaling pathways based on differential gene expression of each subcluster versus all other subclusters. The PPAR signaling pathway for MARCO+ macrophage subcluster 5 and predicted activation (orange) or inhibition (blue) is shown.

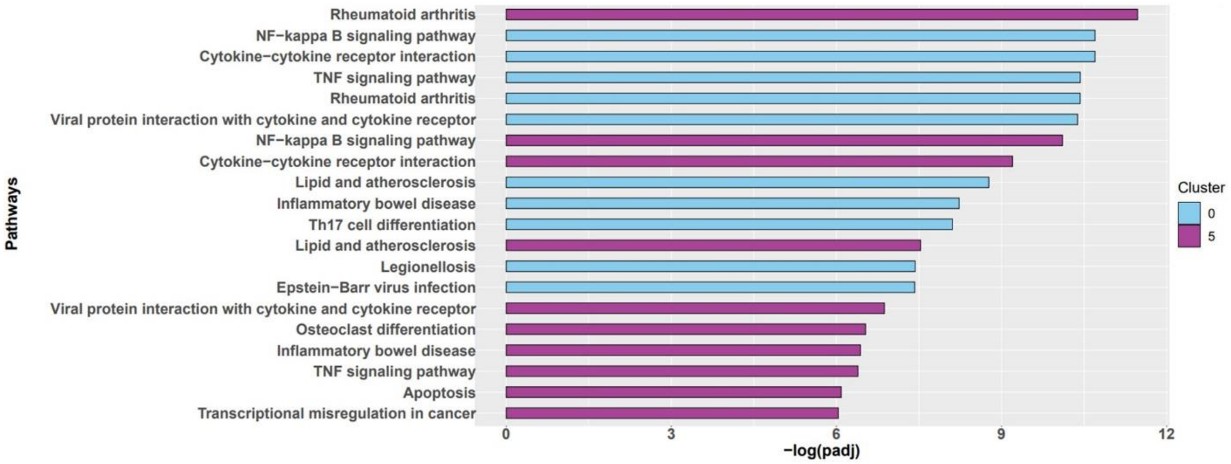

Supplementary Figure S7. Top significantly altered pathways in macrophage subclusters 0 and 5. The top 10 significantly altered KEGG pathways for TREM2+ (subcluster 0, blue) and MARCO+ (subcluster 5, purple) macrophages based on differential gene expression with other macrophage subclusters are shown. The -log(adjusted p-value) is provided for each pathway, ordered based on significance level.

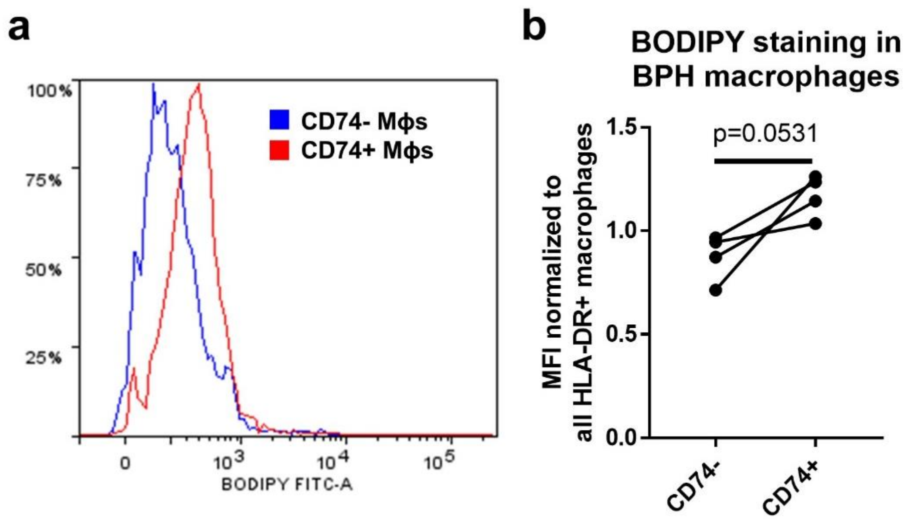

Supplementary Figure S8. Human prostate transition zone tissues were digested and stained for flow cytometry analysis. BODIPY staining intensity was measured in CD74+ versus CD74- subpopulations. **a)** Example histogram of BODIPY median fluorescence intensity (MFI) in CD74+ (red) versus CD74- (blue) macrophage subpopulations. **b)** Quantitation of (a) from 4 individual patients, where the BODIPY MFI was normalized to that of all HLA-DR+ macrophages in each sample. p=0.0531, d=2.70 using a paired, two-tailed t-test.

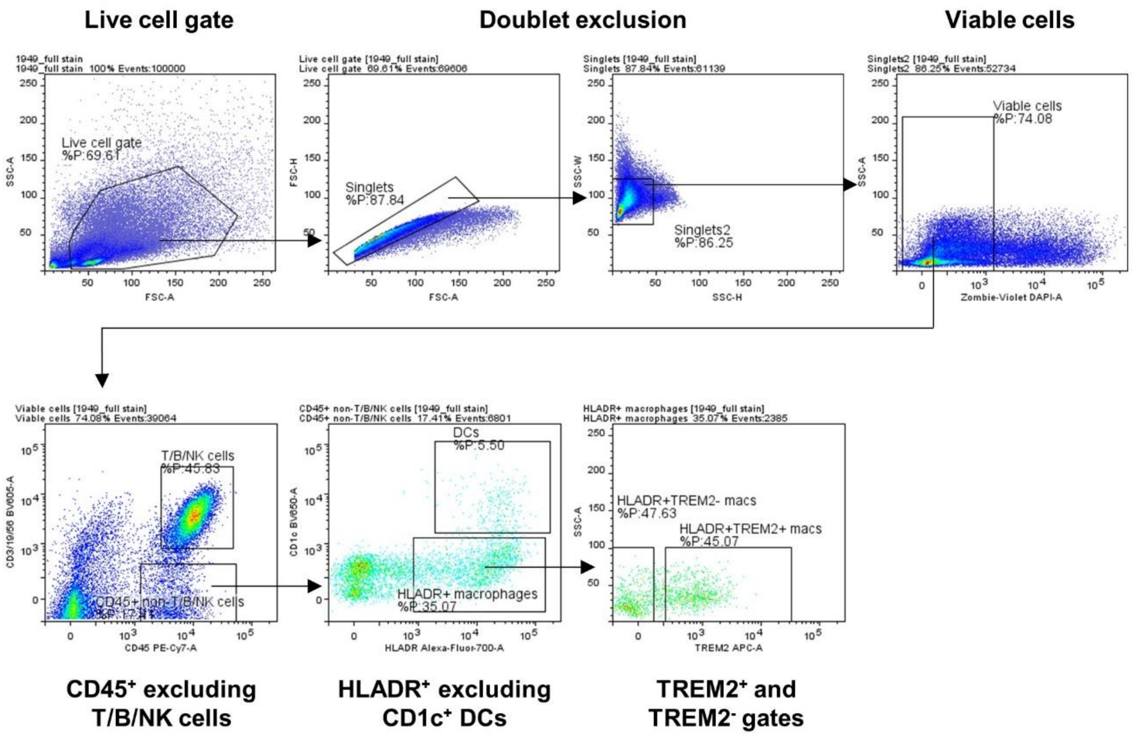

Supplementary Figure S9. Flow gating strategy for the analysis of TREM2+ versus TREM2- macrophages from human BPH tissues. The initial live cell gate was drawn on the SSC vs FSC plot, followed by doublet exclusion gates and viable cell gating on zombie violet-negative events. CD45+ cells that were negative for CD3/19/56 were then evaluated for HLA-DR expression. HLADR+CD1c- macrophages were then separated into TREM2+/- populations, where BODIPY staining was individually evaluated and overlaid for analysis.

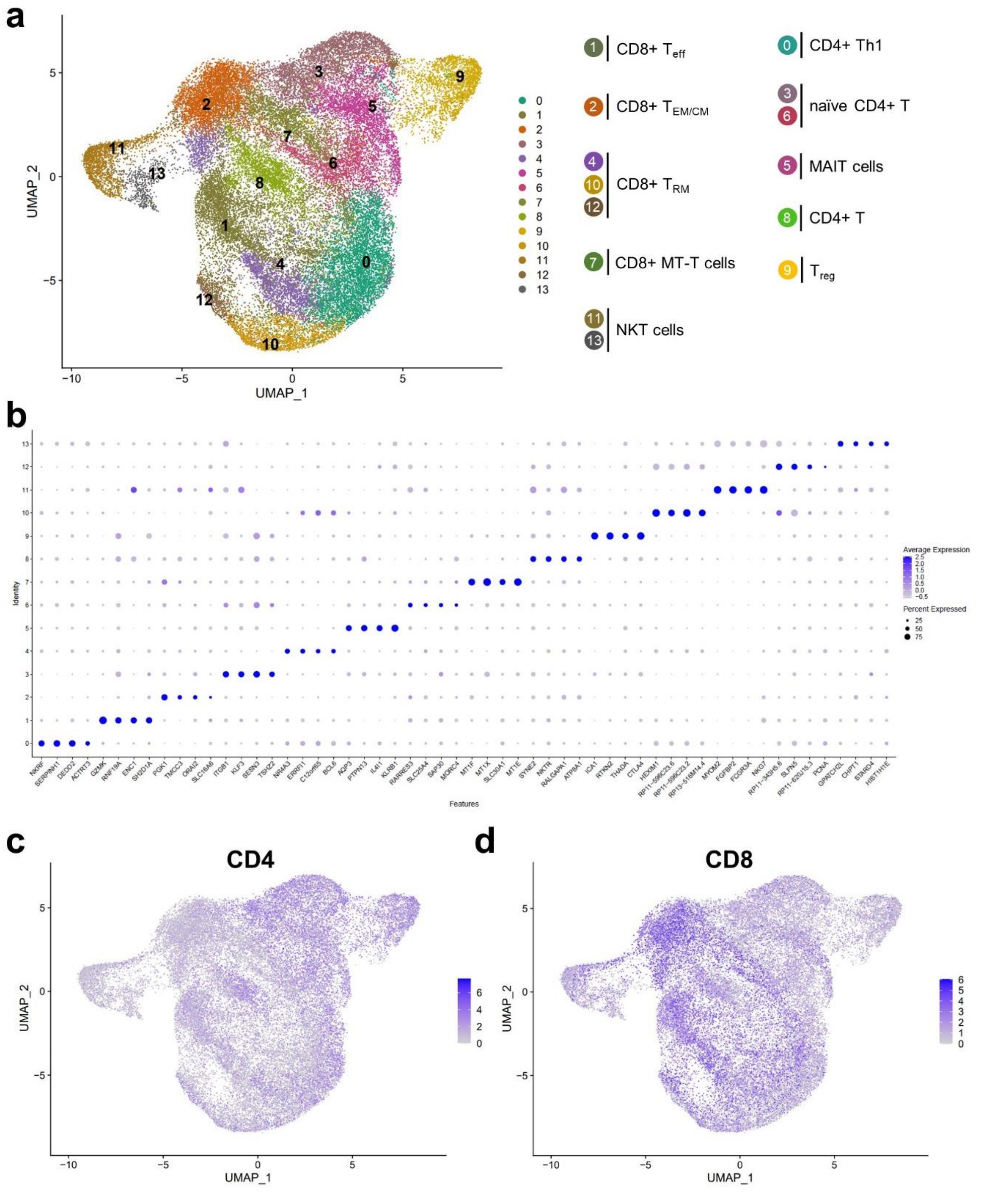

Supplementary Figure S10. T cell subclustering indicates limited changes among subpopulations in large versus small prostates. T cells and CD3+ NK cell clusters from scRNA-seq analysis in Figure 1 were used for subclustering analysis after removal of cells expressing keratin genes or myeloid/B cell specific genes. **a)** UMAP plot indicating 14 T/NKT cell subclusters with putative identity. T_{eff}=effector T cells, $T_{EM/CM}$=effector memory or central memory T cells, T_{RM}=resident memory T cells, MT-T cells = metallothionein-high T cells;

MAIT=mucosal-associated invariant T cells, and T_{reg}=regulatory T cells. **b)** Dot plot indicating the top 4 marker genes for each cluster, where size of the dot indicates the percentage of cells in the cluster expressing the gene and blue intensity indicates average expression of the gene among all cells in the cluster. **c-d)** CITE-seq analysis was conducted on a subset of the 20 patient samples and allowed for the visualization of protein expression in single cells. Feature plots highlight protein expression of c) CD4 and d) CD8, where blue color indicates positive expression and gray color indicates no detectable expression of the indicated protein. Note that cells with gray color may be from samples that did not include CITE-seq analysis.

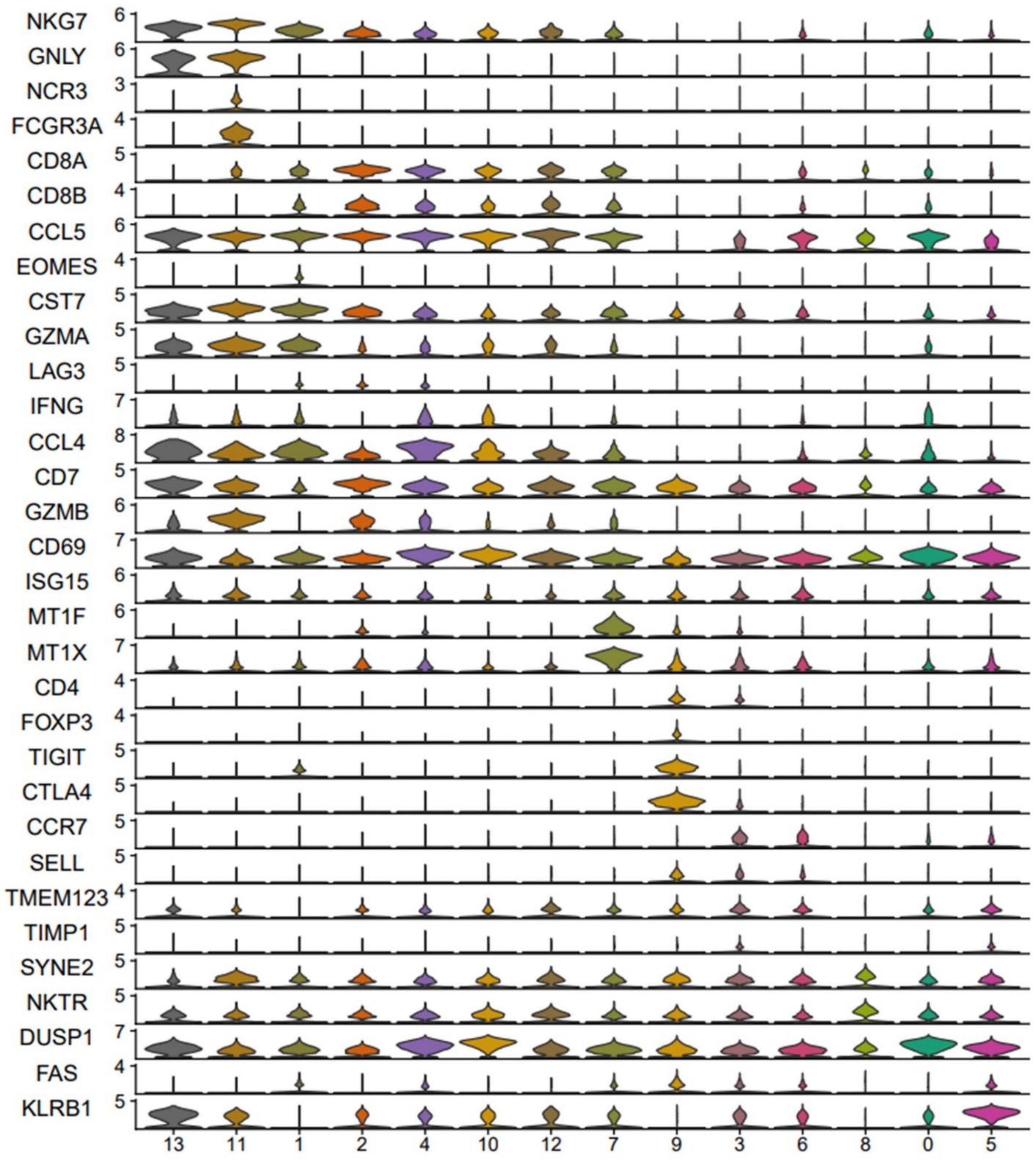

Supplementary Figure S11. Genes used to identify T cell subpopulations. Stacked violin plots show gene expression of the various genes used to identify T cell subclusters. Clusters are arranged by proposed similarity.

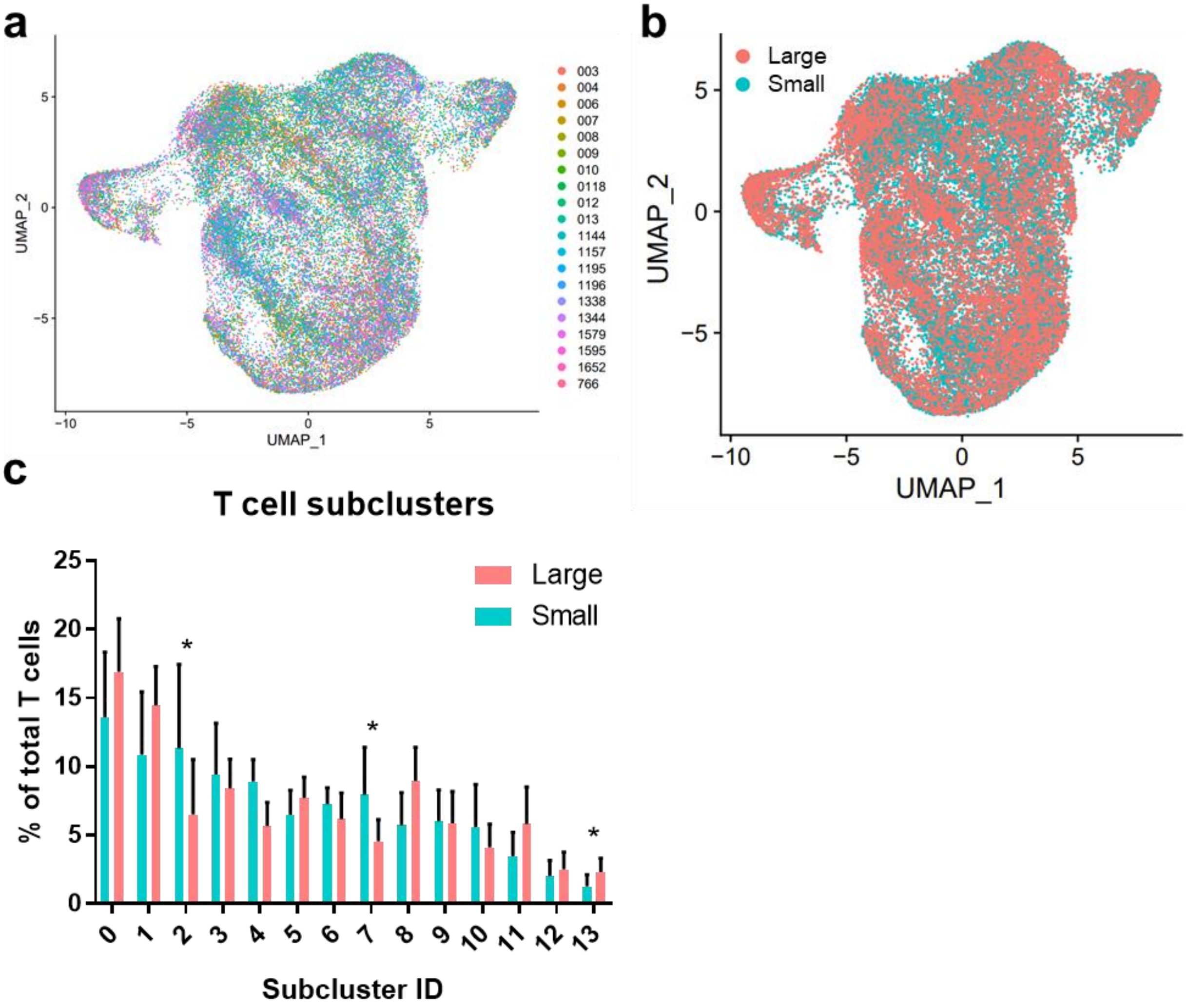

Supplementary Figure S12. T cell subpopulation changes in large versus small prostate TZ tissues. a) UMAP plot of T/NKT cell subclustering analysis, colored to highlight cells from individual patients. **b)** UMAP plot of T/NKT subclusters, colored to highlight cells from small (blue) or large (pink) prostates. **c)** Graph representing the mean +/- SD of the percentage of cells in each T cell subcluster among total T cells between samples in large (pink) versus small (blue) prostates (n=10/group). Asterisks indicate significantly changing populations in large vs small prostate tissues by permutation test (*FDR<0.05).

Supplementary Table S1. Macrophage polarization signature QUSAGE results. BPH macrophage subclusters were determined to fit the M1 or M2 signature if the results indicated a positive log(fold-change) and p-value/FDR<0.01.

Macrophage Subcluster	Pathway Name	log.fold.change	p-value	FDR
0	M1	-0.237196696	0	0
0	M2	-0.14040988	0	0
1	M1	-0.114730529	0	0
1	M2	-0.010223622	0.124533634	0.124533634
2	M1	0.105220036	0	0
2	M2	0.039806782	6.23E-08	6.23E-08
3	M2	-0.135438606	0	0
3	M1	-0.070256232	0	0
4	M1	0.031018465	0.000184514	0.000369028
4	M2	0.016425192	0.047234392	0.047234392
5	M1	-0.158382662	0	0
5	M2	0.017284076	0.019256193	0.019256193
6	M1	-0.11920269	0	0
6	M2	0.141695963	0	0
7	M1	0.135516247	0	0
7	M2	-0.0290059	0.003730118	0.003730118
8	M2	0.135956371	0	0
8	M1	0.299010676	0	0
9	M1	0.429599238	0	0
9	M2	-0.059009949	7.04E-09	7.04E-09
10	M1	-0.319631351	0	0
10	M2	0.402006665	0	0
11	M2	-0.078696317	0	0
11	M1	0.119197828	0	0
12	M1	0.312103101	0	0
12	M2	-0.038738584	0.00106984	0.00106984
13	M1	1.010340694	0	0
13	M2	-0.018382067	0.265059691	0.265059691

Supplementary Table S2. Antibodies used for all studies. Table indicates the antibody target, clone (if monoclonal), manufacturer product number, and dilution used for all antibodies used in these studies. The RRID indicates the antibody identification number on antibodyregistry.org, where available.

Application	RRID	Antibody Target	Clone	Species	Isotype	Company	Ref. #	Antibody Dilution
FACS	AB_2564156	CD45-PE	HI30	Mouse	IgG1,k	Biolegend	304058	1:20
FACS	N/A	EpCAM-APC	9C4	Mouse	IgG2b,k	Biolegend	324208	1:20
FACS	AB_2563247	CD200-PE/Cy7	OX-104	Mouse	IgG1,k	Biolegend	329212	1:20
Flow	AB_314394	CD45-FITC	HI30	Mouse	IgG1,k	Biolegend	304006	1:20
Flow	AB_830644	CD11b-PE/Cy7	ICRF44	Mouse	IgG1,k	Biolegend	301322	1:20
Flow	AB_314248	CD19-APC/Cy7	HIB19	Mouse	IgG1,k	Biolegend	302218	1:20
Flow	AB_314066	CD3-APC	UCHT1	Mouse	IgG1,k	Biolegend	300412	1:20
Flow	AB_314076	CD4-PE	RPA-T4	Mouse	IgG1,k	Biolegend	300508	1:20
Flow	AB_2561942	CD8-BV510	RPA-T8	Mouse	IgG1,k	Biolegend	301048	1:20
Flow	N/A	TREM2-APC	237920	Rat	IgG2B	R&D Systems	FAB17291A	1:20
Flow	AB_2564379	CD3-BV605	UCHT1	Mouse	IgG1,k	Biolegend	300459	1:50
Flow	AB_2562014	CD19-BV605	HIB19	Mouse	IgG1,k	Biolegend	302243	1:50
Flow	AB_2565855	CD56-BV605	5.1H11	Mouse	IgG1,k	Biolegend	362537	1:50
Flow	AB_314404	CD45-PE/Cy7	HI30	Mouse	IgG1,k	Biolegend	304016	1:50
Flow	AB_493771	HLADR-AF700	L243	Mouse	IgG2a,k	Biolegend	307626	1:50
Flow	AB_2800865	CD1c-BV650	L161	Mouse	IgG1,k	Biolegend	331541	1:50
Flow	AB_2229059	CD74-PE	LN2	Mouse	IgG1,k	Biolegend	326807	1:50
CITE-seq	AB_2800722	CD3	UCHT1	Mouse	IgG1,k	Biolegend	300477	1:50-1:200
CITE-seq	AB_2800724	CD4	RPA-T4	Mouse	IgG1,k	Biolegend	300565	1:50-1:200
CITE-seq	AB_2800729	CD8a	RPA-T8	Mouse	IgG1,k	Biolegend	301069	1:50-1:200
CITE-seq	AB_2800731	CD11b	ICRF44	Mouse	IgG1,k	Biolegend	301357	1:50-1:200
CITE-seq	AB_2800740	CD19	HIB19	Mouse	IgG1,k	Biolegend	302263	1:50-1:200
CITE-seq	AB_2814309	CD56 (NCAM)	5.1H11	Mouse	IgG1,k	Biolegend	362561	1:50-1:200
IF	AB_2762383	TREM2	9H4L26	Rabbit	IgG	ThermoFisher	702886	1:100
IF/IHC	AB_2314148	CD68	KP1	Mouse	IgG1,k	Dako/Agilent	M0814	1:100
IF	AB_2534091	Goat anti-mouse AF594	Polyclonal	Goat	IgG	Invitrogen	A11032	1:2000
IF	N/A	Goat anti-rabbit AF488	Polyclonal	Goat	IgG	ThermoFisher	A27034	1:4000

Supplementary Table S3. Run metrics for scRNA-seq of CD45+ cells from human prostate transition zone. Table includes the run metrics from each scRNA-seq sample, including 10 small and 10 large samples. The metrics indicate high quality data and that the average number of cells captured approached the intended number of 5000 cells.

Sample	Number of Reads	% Q30 Bases in Read	% Reads Mapped	Estimated Number of Cells	Mean Reads per cell	Median Genes per cell
003_small	437,303,375	91.3	96.2	6,388	68,457	1,692
004_small	266,767,655	91.7	96.7	4,163	64,080	1,599
006_small	258,138,447	91.5	96.9	6,427	40,164	1,628
007_small	765,500,750	90.8	96.0	4,427	172,916	1,958
008_small	552,744,091	93.0	98.1	5,053	109,389	1,652
009_small	361,879,675	93.3	96.7	3,659	98,901	1,740
010_small	245,628,503	91.6	96.3	3,750	65,500	1,576
0118_large	291,734,653	93.5	97.1	3,803	76,712	1,801
012_large	297,618,482	90.2	96.7	5,826	51,084	1,526
013_small	393,520,430	92.6	96.7	5,230	75,242	1,476
1144_small	394,479,925	90.0	97.0	6,010	65,637	1,662
1157_large	303,893,852	90.4	96.8	6,816	44,585	1,525
1195_large	455,027,712	92.4	96.6	5,377	84,624	1,591
1196_small	224,350,221	92.7	96.8	4,554	49,264	1,489
1338_large	319,810,557	93.5	96.9	6,453	49,560	1,813
1344_large	284,285,224	93.6	97.0	4,245	66,969	1,475
1579_large	363,290,890	91.9	96.0	3,503	103,709	1,549
1595_large	344,446,483	91.4	96.5	6,430	53,569	779
1652_large	323,447,472	91.8	96.7	4,269	75,767	1,552
766_large	368,324,247	90.8	95.6	4,076	90,364	1,335
